# A Post-Processing Algorithm for miRNA Microarray Data

**DOI:** 10.3390/ijms21041228

**Published:** 2020-02-12

**Authors:** Stepan Nersisyan, Maxim Shkurnikov, Andrey Poloznikov, Andrey Turchinovich, Barbara Burwinkel, Nikita Anisimov, Alexander Tonevitsky

**Affiliations:** 1 Faculty of Mechanics and Mathematics, Lomonosov Moscow State University, Leninskie Gory 1, 119991 Moscow, Russia; 2 P.A. Hertsen Moscow Oncology Research Center, Branch of National Medical Research Radiological Center, Ministry of Health of the Russian Federation, Second Botkinsky lane 3, 125284 Moscow, Russia; mshkurnikov@gmail.com; 3 National Medical Research Radiological Center, Ministry of Health of the Russian Federation, 249036 Obninks, Russia; andrey.poloznikov@gmail.com; 4 School of Biomedicine, Far Eastern Federal University, 690922 Vladivostok, Russia; rectorat@dvfu.ru; 5 Molecular Epidemiology C080, German Cancer Research Center, 69120 Heidelberg, Germany; a.turchinovich@dkfz.de (A.T.); B.Burwinkel@dkfz-heidelberg.de (B.B.); 6 SciBerg e.Kfm, 68309 Mannheim, Germany; 7 University Hospital Heidelberg, 69120 Heidelberg, Germany; 8 Faculty of Biology and Biotechnologies, Higher School of Economics, 117312 Moscow, Russia; tonevitsky@mail.ru; 9 Shemyakin-Ovchinnikov Institute of Bioorganic Chemistry RAS, 117997 Moscow, Russia

**Keywords:** miRNA microarrays, miRNome of breast cancer, TCGA

## Abstract

One of the main disadvantages of using DNA microarrays for miRNA expression profiling is the inability of adequate comparison of expression values across different miRNAs. This leads to a large amount of miRNAs with high scores which are actually not expressed in examined samples, i.e., false positives. We propose a post-processing algorithm which performs scoring of miRNAs in the results of microarray analysis based on expression values, time of discovery of miRNA, and correlation level between the expressions of miRNA and corresponding pre-miRNA in considered samples. The algorithm was successfully validated by the comparison of the results of its application to miRNA microarray breast tumor samples with publicly available miRNA-seq breast tumor data. Additionally, we obtained possible reasons why miRNA can appear as a false positive in microarray study using paired miRNA sequencing and array data. The use of DNA microarrays for estimating miRNA expression profile is limited by several factors. One of them consists of problems with comparing expression values of different miRNAs. In this work, we show that situation can be significantly improved if some additional information is taken into consideration in a comparison.

## 1. Introduction

**Figure 1:**
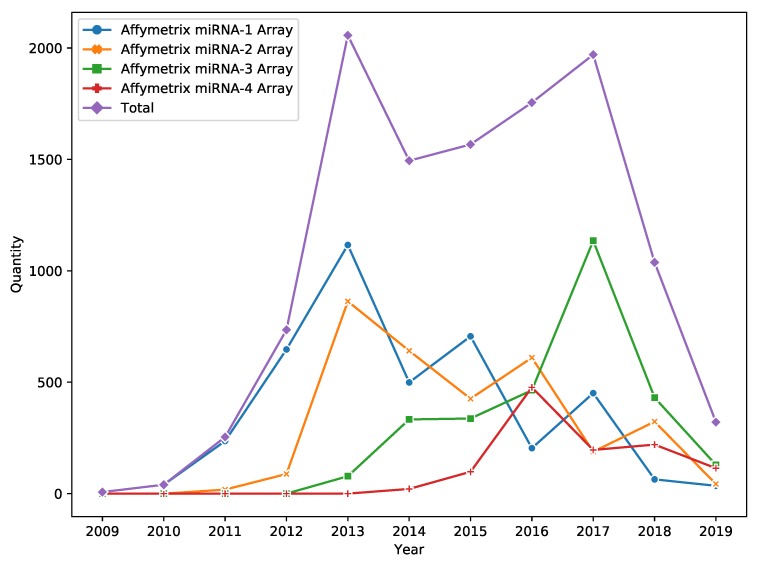
Number of Gene Expression Omnibus (GEO) accessions corresponding to Affymetrix miRNA Array data.

DNA microarray is a popular high throughput technology for miRNA expression profiling. Despite the fact that technology passed the peak of its popularity in 2017, it is still widely used in miRNA research (e.g., see Figure 1 for information about the yearly amount of Affymetrix miRNA Array data published in Gene Expression Omnibus (GEO) [1]).

Usually, miRNA microarray analysis can be summarized as follows [2,3,4]. First, miRNA is extracted from sample, reverse-transcribed and marked with fluorescent label. Then, labeled miRNAs are hybridized on the array. The final step consists of generating an image using laser-induced fluorescent imaging. The key mechanism behind the described procedure is that the amount of fluorescence measured at each probe is proportional to the amount of hybridized miRNA sequences present in the sample considered. As a result of such study, raw relative concentration (intensity) files are generated. These files represent a table where rows correspond to known miRNAs and columns correspond to examined samples; cells of a table contain expression values. Raw expression tables cannot be used both for intra-miRNA comparisons (i.e., comparing expression levels of some miRNA across samples) and inter-miRNA comparisons (i.e., comparing expression levels of different miRNAs in some sample) because of different scales and relative nature of intensity values.

Intra-miRNA comparisons play a key role in miRNA studies, e.g., for differential expression analysis. Several normalization methods which allow for performing adequate comparison of miRNA expression levels were proposed and now always used as a necessary preprocessing step. The most advanced techniques based on *loess* model construction show really good results in terms of consistency of normalized expression values with results from polymerase chain reaction (PCR), see [5,6] for a detailed survey.

However, all of these methods do not help to overcome the problem of inter-miRNA comparisons—the fact that obtained expression values cannot be directly compared between different miRNAs. The reason for this phenomenon is linked to the fact that microarray analysis does not directly provide information on the absolute level of expression of a particular miRNA. Above-mentioned normalization methods perform scaling of raw intensity values by focusing on intra-miRNA expression variability, thus making expression values hardly comparable between different miRNAs (see, e.g., [7]). In particular, a large number of miRNAs receive high absolute expression values which contradicts with the fact that the number of highly expressed miRNAs in human tissues is relatively small, usually a few dozen (see, e.g., [8]). Moreover, the indicated problem can be observed while comparing miRNA expression profiles obtained by different microarray platforms, see [9]: a particular miRNA in a sample can have significantly different expression estimates when analysis is performed by two different microarray platforms. Despite the fact that the results of miRNA microarray analysis can be validated by more accurate methods like PCR, the problem of inter-miRNA comparisons is still challenging since any solution will lead to the possibility of reducing the number of miRNAs that need to be further validated by more accurate techniques.

Thus, results of microarray miRNA expression profiling should be post-processed in order to allow for inter-miRNA comparisons. In this work, we propose a post-processing algorithm which assigns a score (number between 0 and 1) to each miRNA according to the degree of its reliability in analyzed samples. Score calculation procedure is based on three values:

1. Estimated expression values across all samples;
2. MIMAT—chronological accession number taken from the miRBase database [10];
3. Correlation level between expression values of considered miRNA and corresponding pre-miRNA.

The closer the score is to 1, the higher probability that miRNA is really expressed in considered samples.

In order to validate the proposed algorithm, we applied it to the miRNA microarray data derived from 30 breast tumor samples from [11] and compared obtained results with “reference” miRNA-seq data from The Cancer Genome Atlas Breast Invasive Carcinoma (TCGA-BRCA) project [12] derived from 850 breast tumor samples of stages I and II. In addition, we analyzed 12 blood plasma samples using miRNA-seq and miRNA microarrays in order to characterize false positive miRNAs.

## 2. Results

### 2.1. miRNA Profile of TCGA Samples

**Figure 2:**
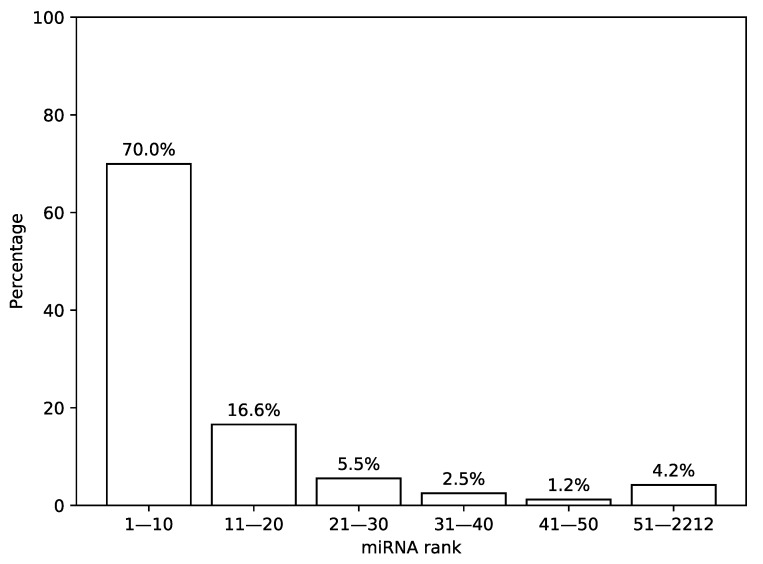
Distribution of expression values in The Cancer Genome Atlas (TCGA) miRNA-seq data. In this figure, a percentage of total miRNA expression covered by the first *n* most highly expressed miRNAs according to the TCGA miRNA-seq data are shown.

First, we analyzed the distribution of the most highly expressed miRNAs in TCGA miRNA-seq samples. For that, we sorted a list of miRNAs according to a median expression value calculated across all samples and analyzed the results by calculating percentage of total expression covered by first *n* miRNAs from the list, see Figure 2. It can be seen that 30 miRNAs with the highest expression values cover more than 92% of the whole distribution mass while the remaining 8% are spread across the next 2182 miRNAs.

**Figure 3:**
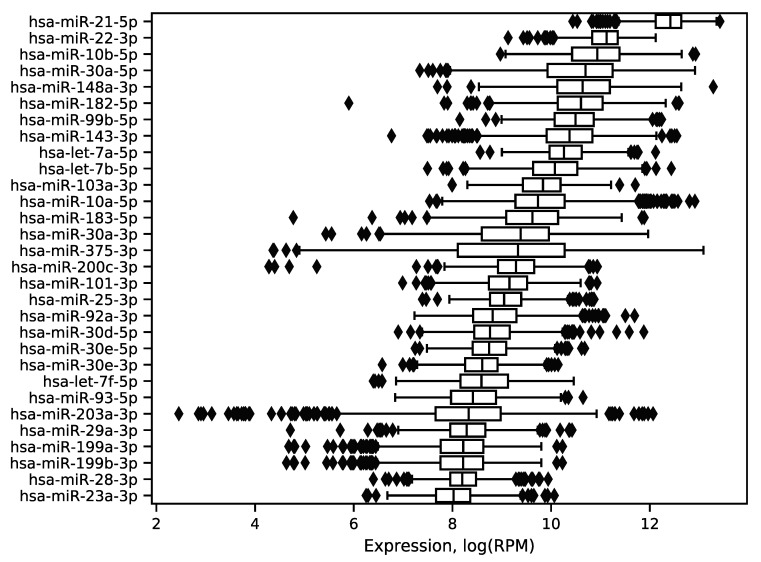
Distribution of the 30 best expressed miRNA expressions in the TCGA miRNA-seq data. The logarithm base 2 was utilized.

**Figure 4:**
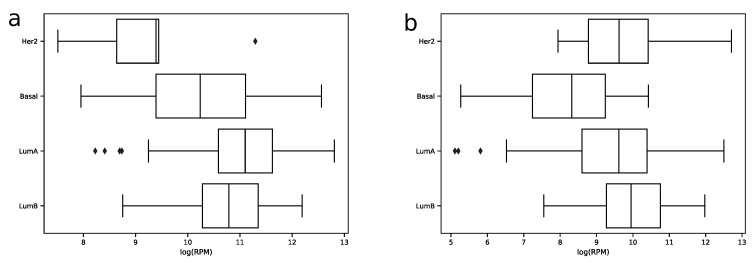
Distribution of hsa-miR-30-5p (**a**) and hsa-miR-375-3p (**b**) $\log_{2}$-transformed expression values in samples of four breast cancer molecular subtypes in the TCGA miRNA-seq data.

In order to explore expression distribution of the first 30 miRNAs, a boxplot was constructed, see Figure 3. In can be seen that almost all miRNA expressions despite outliers are lowly dispersed across samples except some miRNAs like hsa-miR-30a-5p or hsa-miR-375-3p. Interestingly, such variability can be explained in terms of molecular subtypes of breast cancer [13], namely, for these two miRNAs expression distributions are much less dispersed in each of four subtypes (luminal A, luminal B, HER2 overexpression and basal), see Figure 4. The results of formal comparison with a two-sided Mann–Whitney U-test are summarized in Table 1.

### 2.2. Using Score Instead of Raw Expression Significantly Reduces the Number of False Positives

**Figure 5:**
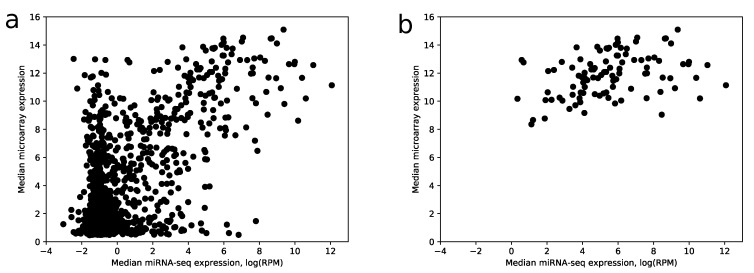
Median miRNA-seq expression vs. median microarray expression. (**a**) scatter plot showing joint distribution of median expression values for all miRNAs from microarray and miRNA-seq data; (**b**) same plot with miRNAs filtered by a 0.75 score value threshold. The logarithm base 2 was utilized.

Despite the fact that the most highly expressed miRNAs according to miRNA-seq have relatively large expressions in the microarray data (28 of 30 top miRNAs have median expression larger than the upper quartile taken from the whole data), there is a big amount of miRNAs with a large microarray and low miRNA-seq expressions, i.e., false positives, see Figure 5a.

The proposed algorithm was applied to the results of microarray expression profiling data. To show the increase of specificity after score calculation, we analyzed mutual intersections of two sets:

1. miRNAs with score greater than 0.75;
2. miRNAs with median expression level greater than 10 reads per million (RPM).

The comparison shows that 104 out of 114 miRNAs with the best scores have median RPM value greater than 10. If use raw expression values instead of scores (with the same number of miRNAs), the number of miRNAs with RPM less than 10 increases by 2.5 times. Note that scoring coefficients (see Section 4) were selected in the way to maximize this ratio. In addition, the increase of specificity can be observed in Figure 5b.

To study sensitivity of the algorithm, we compared scores of miRNAs with median RPM greater than 10 with the remaining ones. The first group had mean score 0.69 with standard deviation 0.18, while the second one had mean score 0.33 with standard deviation 0.16. Application of the Mann–Whitney U test showed that score value within the first group was significantly greater compared to the second group ($p<10^{-10}$).

### 2.3. Number of False Positive miRNAs Increases with MIMAT Number

**Figure 6:**
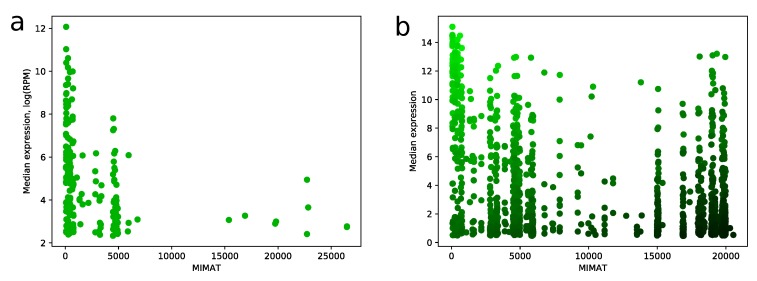
MIMAT number vs expression level. (**a**) scatter plot showing relation between MIMAT number and $\log_{2}$-transformed median expression value for all miRNAs from TCGA miRNA-seq data with RPM value greater than 10; (**b**) the same plot for all miRNAs from microarray data. Color of point indicates score value: green points correspond to high scores and black points correspond to low scores.

MIMAT is a unique chronological accession number of miRNA from the miRBase database. We noticed a strong dependence between MIMAT number and expression level of miRNA in miRNA-seq samples. Namely, distribution of MIMAT numbers on the set of miRNAs with median RPM value greater than 10 is concentrated on a range between 0 and 5000, see Figure 6a. Similar results can be found e.g., in [7,14]. This fact gave us a strong ground to consider MIMAT number as a factor in the scoring algorithm and be careful with assigning high scores to miRNAs with big MIMAT value. The resulting trade-off between score and MIMAT number is illustrated in Figure 6b.

### 2.4. Consideration of miRNA—Pre-miRNA Correlation Increases Sensitivity of the Algorithm

Correlation between miRNA and corresponding pre-miRNA expression levels was added as a factor in the scoring algorithm for increasing its sensitivity: slight promotion of miRNAs with a high correlation level can help to identify not very high expressed miRNAs which are presented in considered samples. For example, such miRNAs as hsa-miR-18a-5p, hsa-miR-30a-3p, hsa-miR-194-5p, hsa-miR-532-5p, hsa-miR-652-3p have RPM value greater than 10 in the sequencing data, but their score is lower than 0.75 if correlation factor is removed from the scoring algorithm.

### 2.5. False Positive miRNAs Tend to Have Higher GC-Content and Appear in the Genome More Frequently Compared to Others

In order to investigate why we observe such a big number of false positive miRNAs in microarray data, we took 12 human blood plasma samples and analyzed their miRNome using miRNA-seq and Affymetrix miRNA 4.0 arrays. First, we calculated GC-content for all sequences of utilized microarray probe sets (in our case, these sequences were the same as a corresponding miRNA sequence). Then, we identified 212 miRNAs which are highly expressed in microarray data (top 10% median expression values) and are not expressed in miRNA-seq data (joint distribution is illustrated in Supplementary Figure S1). Comparison of 212 corresponding GC-contents with all remaining 2319 GC-contents using a Mann–Whitney U test resulted in the statistically significant fact that false positive miRNAs tend to have larger GC-content value ($p<10^{-10}$). The fact that GC base pair has three hydrogen bonds, while AT has two, which makes GC interactions more powerful. Thus, analyzed sequences have more potential to base-pair with a complementary one even with several mismatches.

After that, we mapped all miRNA sequences to the human genome allowing up to two mismatches and outputting all mapped sites. As a result, we obtained the number of genomic occurrences for each miRNA. Finally, we show that this number is greater for considered 212 false positive miRNAs compared to all others (Mann–Whitney U test, $p<10^{-7}$).

## 3. Discussion

The algorithm proposed performs scoring of miRNAs in the results of microarray analysis. We validate its performance by demonstrating concordance between miRNAs with high scores with highly expressed miRNAs according to miRNA-seq data. The importance of each algorithm feature was justified by the following arguments:

- There is a strong negative dependence of miRNA expression level with its chronological MIMAT accession number in the TCGA miRNA-seq data;
- The number of false positive miRNAs in microarray data (i.e., miRNAs with high expression values which are actually low expressed in analyzed samples) increases with MIMAT number;
- The use of miRNA–pre-miRNA correlations helps to increase sensitivity of the algorithm.

In order to better understand miRNA false-positivity phenomena, we studied paired microarray and miRNA-seq blood plasma samples. We showed that false positive miRNAs have significantly greater GC-content values and that their sequences appear in the human genome significantly more frequently compared to remaining ones. These results provide theoretical insights about mechanisms of miRNA microarray experimental strategy.

The scope of the proposed algorithm is not limited just to a sorting list of miRNAs based on their expression score. For example, it can be used as an additional step in differential expression analysis: miRNAs with low scores can be marked as low confidence to prevent generating a large number of false positives in the analysis results. Another advantage of the algorithm is a platform independence, i.e., it can be applied to any miRNA microarray data regardless of technology used.

Unfortunately, the method cannot be directly applied in the case of a low number of samples because of the inability of adequate calculation of Spearman correlation between pre-miRNA and miRNA expressions. One of the possible solutions include setting zero weight to the third argument of the scoring function. However, this approach can significantly decrease sensitivity of the algorithm.

## 4. Materials and Methods

### 4.1. miRNA Scoring Algorithm

The miRNA scoring algorithm consists of matching a score from the range [0,1] to each individual miRNA. First, the following three values are determined:

1. Median expression value *m* taken across all considered samples;
2. MIMAT miRNA accession number *N* from the miRBase database;
3. Spearman correlation *c* between expression values of miRNA and expression values of corresponding miRNA precursor.

Then, three obtained numbers are scaled to interval [0,1]:

(1) $$A=\frac{m}{m_{max}},B=1-\frac{N}{N_{max}},C=\frac{c+1}{2},$$

where all maxima are taken across all miRNAs. It can be seen that values *A* and *C* grow with median and correlation values, and *B* decreases with MIMAT number growth.

We define a score as a weighted combination of values *A*, *B*, *C* with weights 0.5, 0.3 and 0.2, respectively. Utilized weight values were chosen in order to maximize specificity of the algorithm, see the Results section for details. The obtained score value lies in interval [0,1] as a weighted combination of numbers from the same interval. Note that using a median instead of mean and Spearman correlation instead of Pearson’s one increases the robustness of the algorithm to outliers.

### 4.2. Utilized miRNA Microarray Data

In this study, we utilized miRNA microarray data derived from 30 breast tumor samples obtained by Affymetrix miRNA 3.0 arrays. Details of experiments can be found in work [11].

### 4.3. Utilized TCGA Data

Processed TCGA-BRCA miRNA-seq data was downloaded from https://portal.gdc.cancer.gov in the format of an miRNA expression table.

### 4.4. Blood Plasma Processing

Twelve samples of male volunteers were obtained in the National Medical Research Radiological Center after they signed informed consent to participate in the study. Local Ethics committee of the National Medical Research Radiological Center granted Ethical approval to carry out the study within its facilities (#2018-11, 27 December 2018).

RNA was isolated by a modified protocol [15]: 400 μL sample of plasma was added to 1.2 mL Qiazol Lysis Reagent (Qiagen, Germany), thoroughly mixed for 15 s, and incubated for 10 min at room temperature for complete dissociation of nucleoprotein complexes. After that, 1.6 μL of Ambion GlycoBlue Coprecipitant (15 mg/mL; Thermo Fisher Scientific, USA) was added to the mixture to increase the efficiency of RNA precipitation during isolation on columns. Then, 320 μL chloroform was added to the mixture, the sample was stirred for 45 s and incubated at room temperature for 5 min; then, the mixture was centrifuged at 16,000× *g* and 4 °C for 20 min and total RNA was isolated using an miRNeasy Serum reagent/Plasma Kit (Qiagen, Germany) according to the manufacturer’s protocol.

### 4.5. Microarray Analysis

Analysis of miRNA in RNA samples isolated from plasma was performed on GeneChip miRNA 4.0 microchips (Affymetrix) according to the manufacturer’s protocol. The results of microchip analysis were preprocessed by the RMA method using Affymetrix Expression Console software (version 1.4.1.46) at RMA+DABG-NO-NORM-Human-only setting. The microarray CEL files for blood plasma samples have been deposited in the GEO database (accession GSE138411).

### 4.6. miRNA Sequencing and Quantification

The CATS Small RNA-seq (Diagenode, Belgium) kit was used to prepare miRNA libraries. Samples were sequenced on the Illumina HiSeq 2000 V4. The miRNA-seq FASTQ files for blood plasma samples have been deposited in the Sequence Read Archive (SRA) (accession PRJNA575803).

miRNA-Seq data analysis and preparation were conducted by the miRDeep2 pipeline [16]. The $3^{\prime }$ adaptor sequence was removed and reads with the trimmed length <18 bp were discarded using Cutadapt v2.7 [17]. Read quality control was performed with FastQC v0.11.9 (Supplementary File S2). Trimmed and filtered reads were mapped with bowtie (v1.1.1) [18] to the reference genome (GRCh38.p12) allowing a maximum of one mismatch. Calculation of the raw read counts and reads per million (RPM) values was performed using miRDeep2 (miRBase v22.0 was utilized).

### 4.7. Computer Code and Software

The proposed algorithm was implemented in Python 3 programming language and available under MIT License at https://github.com/s-a-nersisyan/PP_miRNA_arrays.

## Figures and Tables

**Table 1:** Results of the Mann–Whitney U-test applied to expressions of miRNAs in The Cancer Genome Atlas (TCGA) miRNA-seq data.

Class 1	Class 2	*p*-Value for hsa-miR-30a-5p	*p*-Value for hsa-miR-375-3p
Luminal A	Luminal B	0.18	0.11
Luminal A	Basal	$1.5\times 10^{-5}$	$7.9\times 10^{-6}$
Luminal A	Her2	$7.8\times 10^{-3}$	0.77
Luminal B	Basal	$3.5\times 10^{-2}$	$1.2\times 10^{-5}$
Luminal B	Her2	$2.2\times 10^{-2}$	0.79
Basal	Her2	0.12	$4.6\times 10^{-2}$

## Supplementary Materials

Supplementary materials can be found at https://www.mdpi.com/1422-0067/21/4/1228/s1.
