# Supplementary material for: A Post-Processing Algorithm for miRNA Microarray Data
Source: Int J Mol Sci. 2020 Feb 12;21(4):1228. doi: 10.3390/ijms21041228 (PMC7072892; doi:10.3390/ijms21041228)

Figure 1. Scatter plot showing joint distribution of median expression values for all miRNAs from microarray and miRNA-seq data of 12 blood plasma samples. The logarithm base 2 was utilized.

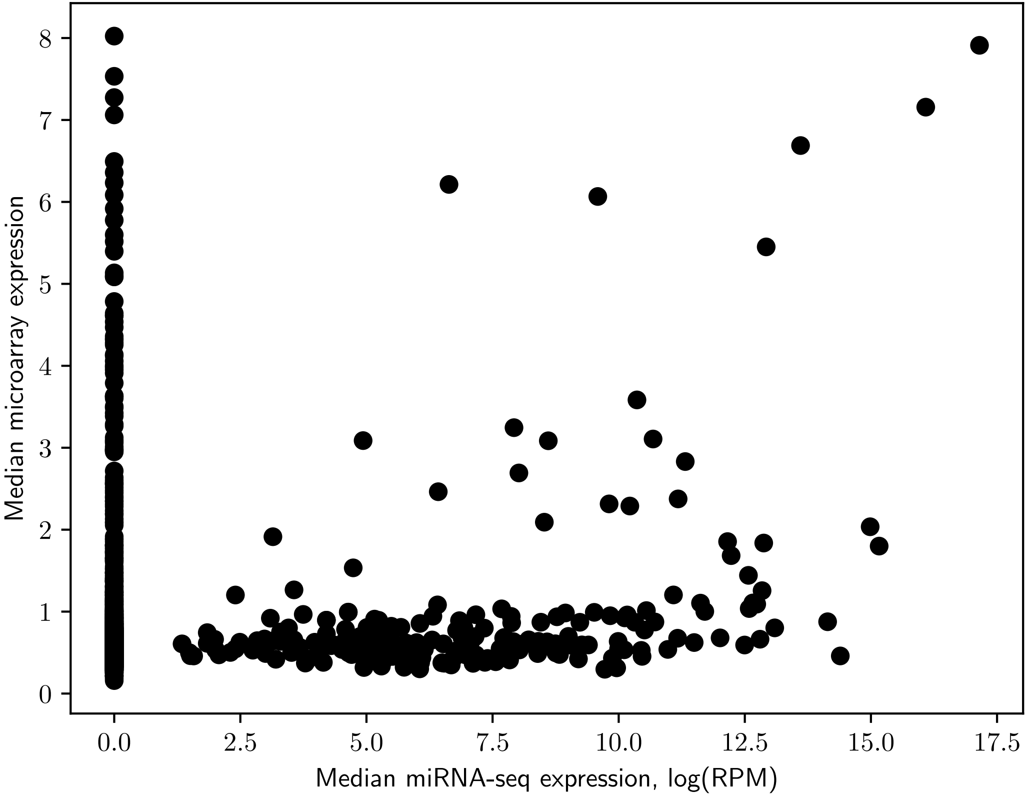

Supplement: Supplementary file 1 [file ijms-21-01228-s001.zip › s1.pdf]
