# Supplementary material for: A Post-Processing Algorithm for miRNA Microarray Data
Source: Int J Mol Sci. 2020 Feb 12;21(4):1228. doi: 10.3390/ijms21041228 (PMC7072892; doi:10.3390/ijms21041228)
Supplement: Supplementary file 1 [file ijms-21-01228-s001.zip › s2.html]

98c\_SS.fastq FastQC Report


# 98c\_SS.fastq FastQC Report

FastQC Report

Sat 1 Feb 2020  
103pc\_SS.fastq

## Summary

- Basic Statistics
- Per base sequence quality
- Per tile sequence quality
- Per sequence quality scores
- Per base sequence content
- Per sequence GC content
- Per base N content
- Sequence Length Distribution
- Sequence Duplication Levels
- Overrepresented sequences
- Adapter Content

## Basic Statistics

| Measure | Value |
| --- | --- |
| Filename | 103pc\_SS.fastq |
| File type | Conventional base calls |
| Encoding | Sanger / Illumina 1.9 |
| Total Sequences | 9724032 |
| Sequences flagged as poor quality | 0 |
| Sequence length | 15-48 |
| %GC | 57 |

## Per base sequence quality

## Per tile sequence quality

## Per sequence quality scores

## Per base sequence content

## Per sequence GC content

## Per base N content

## Sequence Length Distribution

## Sequence Duplication Levels

## Overrepresented sequences

| Sequence | Count | Percentage | Possible Source |
| --- | --- | --- | --- |
| GGGCCGCCGGTGAAATACCACTAC | 574860 | 5.911745251352525 | No Hit |
| GGGTCGGGGCGGCGGC | 94964 | 0.9765907804499204 | No Hit |
| CCCCCGCGGGGGCGCGCCGGC | 74632 | 0.7675005594387184 | No Hit |
| GGGGGCGGGGAGCGGT | 73480 | 0.7556536218720794 | No Hit |
| GAGAGAGGGGCCCGT | 61168 | 0.6290394766286248 | No Hit |
| GGCTGGTCCGATGGTAGTGGGTTATCAGAAC | 49116 | 0.5050991193776408 | No Hit |
| AACCCGGCGGCGGGT | 48811 | 0.5019625603864734 | No Hit |
| CCCCGGGGAGCCCGGCGGGC | 44265 | 0.4552124057181218 | No Hit |
| GGGGGGAGAGAAGGGT | 41807 | 0.42993482538930355 | No Hit |
| CCCCGCGGGGGCGCGCCGGC | 34991 | 0.3598404447866893 | No Hit |
| GGCCGCCGGTGAAATACCACTAC | 33006 | 0.3394271018441733 | No Hit |
| CCCCACAACCGCGCTTGACTAGC | 32377 | 0.3329585916623886 | No Hit |
| CCCCACAACCGCGCTTGACTAGCTT | 32339 | 0.33256780726348906 | No Hit |
| GGCGGGGCGCGGGAC | 32054 | 0.3296369242717424 | No Hit |
| CCCCACAACCGCGCTTGACTAGCTTGCTGTTT | 26969 | 0.27734380141899984 | No Hit |
| GGGCCGCCGGTGAAATACCACTACG | 26737 | 0.27495795982571836 | No Hit |
| CCCGGGGCCGAGGGAGC | 26240 | 0.26984691124011106 | No Hit |
| GGGCGCTGACCCCCT | 24748 | 0.2545034816833182 | No Hit |
| GGCCCCGCCGGGGTCGGC | 24203 | 0.24889881069910094 | No Hit |
| CCCCACTGCTAAATTTGACTGGCT | 23859 | 0.2453611835090629 | No Hit |
| ACCCCCGCGGGGAAT | 23552 | 0.24220405691795338 | No Hit |
| GGGTCGGGGCGGCGGCGGCGGC | 23458 | 0.24123737972067552 | No Hit |
| GGGGGCGGCGCGCGC | 22547 | 0.23186883794705737 | No Hit |
| GGAGATGGGCGCCGC | 20762 | 0.21351225499874948 | No Hit |
| GGGGGCGGGGAGCGGC | 20477 | 0.21058137200700286 | No Hit |
| GTCCCCCGAAGAGGGGGACGGC | 20382 | 0.20960441100975397 | No Hit |
| GAATCCCCGCCCCGC | 20229 | 0.2080309896141847 | No Hit |
| CCGAGAGAGGGGCCCGT | 20157 | 0.20729055601626978 | No Hit |
| GGGCCGCCGGTGAAATACCACTAT | 19435 | 0.1998656524371783 | No Hit |
| GGCCGGGGGGCGGGCGC | 19189 | 0.19733583764430226 | No Hit |
| GGGTCGGTCGGGCTGGGGCGC | 19019 | 0.19558759164922535 | No Hit |
| AGGGAAGAGCCCAGCGCCGAATCCCCGCCCCGC | 18839 | 0.19373650765443798 | No Hit |
| CGCGAGGGGGGCCCGGGC | 18802 | 0.19335600705550948 | No Hit |
| CCGCGAGGGGGGCCCGGGC | 18633 | 0.19161804486040357 | No Hit |
| AGGGCGCCCTGGAAT | 18195 | 0.1871137404730877 | No Hit |
| CCCCGGTGTCCCCGC | 17988 | 0.18498499387908227 | No Hit |
| GTCTACGGCCATACCACCCTGAACGCGCCCGATCTCGTCTGATCTCGG | 17900 | 0.18408001948163066 | No Hit |
| CCCGCCGGGGTCGGC | 17824 | 0.18329845068383158 | No Hit |
| GAGAGAGGGGCCCGC | 17501 | 0.17997678329318537 | No Hit |
| GAAGCCAGAGGAAACTCTGGT | 17055 | 0.17539020850610118 | No Hit |
| GAGGCGTCCAGTGCGGTAACGCGAC | 16564 | 0.17034086272032012 | No Hit |
| GAAGCCAGAGGAAACTCTGGTGGAGGT | 16538 | 0.17007348392107305 | No Hit |
| GAAGAGGGGGACGGC | 14465 | 0.14875516658110544 | No Hit |
| CCCCACAACCGCGCTTGACTAGCT | 13780 | 0.1417107636009425 | No Hit |
| CCCCGAATCCGGAGTGGCGGAGATGGGCGCCGCGAGGCGTCCAGTGCG | 13243 | 0.1361883630164936 | No Hit |
| GGGAAGGCCCGGCGGGGAAGGT | 13009 | 0.13378195382327002 | No Hit |
| GGCTGGTCCGATGGTAGTGGGTTATC | 13005 | 0.13374081862338585 | No Hit |
| GGAGAAGCCGGCGGGAGC | 12792 | 0.13155036922955415 | No Hit |
| GGCCGGGGGGCGGGC | 12426 | 0.12778649844015322 | No Hit |
| GGCGGGGCGCGGGACATGTGGCGTACGGAAGACCCGC | 12359 | 0.1270974838420935 | No Hit |
| GGGGAGCCCGGCGGGC | 12237 | 0.1258428602456265 | No Hit |
| GGCGGGGCGCGGGACATGTGGCGT | 12231 | 0.12578115744580026 | No Hit |
| GGGCCGCCGGTGAAATACCACTACAG | 12037 | 0.12378610025141834 | No Hit |
| AGCCCCTCTCCGGCC | 11358 | 0.11680340007108163 | No Hit |
| CGACTCTTAGCGGTGGATCACTCGGCTCGTGCGTCGATGAAGAACGCA | 11027 | 0.11339946228066712 | No Hit |
| CGGTCGGGCTGGGGCGC | 11016 | 0.11328634048098567 | No Hit |
| GGGCCGCCGGTGAAAT | 10820 | 0.11127071568666166 | No Hit |
| TGGCCGCCGGTGAAATACCACTAC | 10543 | 0.10842210309468336 | No Hit |
| CCCCCGCGGGGGCGCGCCGGCGT | 10451 | 0.1074759934973476 | No Hit |
| GGGCGGGGCCGGGGGT | 10387 | 0.10681783029920099 | No Hit |
| GGGCCGCCGGTGAAATACCACTACAT | 10152 | 0.1044011373060064 | No Hit |
| GGGGGGAGAGAAGGGC | 9848 | 0.10127486211480999 | No Hit |

## Adapter Content

Produced by FastQC (version 0.11.8)

FastQC Report

Sat 1 Feb 2020  
106b\_SS.fastq

## Summary

- Basic Statistics
- Per base sequence quality
- Per tile sequence quality
- Per sequence quality scores
- Per base sequence content
- Per sequence GC content
- Per base N content
- Sequence Length Distribution
- Sequence Duplication Levels
- Overrepresented sequences
- Adapter Content

## Basic Statistics

| Measure | Value |
| --- | --- |
| Filename | 106b\_SS.fastq |
| File type | Conventional base calls |
| Encoding | Sanger / Illumina 1.9 |
| Total Sequences | 12180869 |
| Sequences flagged as poor quality | 0 |
| Sequence length | 15-48 |
| %GC | 57 |

## Per base sequence quality

## Per tile sequence quality

## Per sequence quality scores

## Per base sequence content

## Per sequence GC content

## Per base N content

## Sequence Length Distribution

## Sequence Duplication Levels

## Overrepresented sequences

| Sequence | Count | Percentage | Possible Source |
| --- | --- | --- | --- |
| GGGCCGCCGGTGAAATACCACTAC | 1171621 | 9.61853378441226 | No Hit |
| GGGCCGCCGGTGAAAT | 78051 | 0.640767091411951 | No Hit |
| GGCCGCCGGTGAAATACCACTAC | 72202 | 0.5927491708514392 | No Hit |
| CCCCACAACCGCGCTTGACTAGCTTGCTGTTT | 69621 | 0.5715602064187703 | No Hit |
| GGCTGGTCCGATGGTAGTGGGTTATCAGAAC | 66728 | 0.5478098483778128 | No Hit |
| CCCCACAACCGCGCTTGACTAGC | 64676 | 0.5309637596463767 | No Hit |
| GGGCCGCCGGTGAAATACCACTACG | 62583 | 0.5137810775241076 | No Hit |
| CCCCCGCGGGGGCGCGCCGGC | 62465 | 0.5128123453261011 | No Hit |
| GGGTCGGGGCGGCGGC | 55026 | 0.45174116887719584 | No Hit |
| GGGCCGCCGGTGAAATACCACTAT | 54221 | 0.4451324449840155 | No Hit |
| GAGAGAGGGGCCCGT | 47672 | 0.3913678079946513 | No Hit |
| AGGGAAGAGCCCAGCGCCGAATCCCCGCCCCGC | 45765 | 0.3757121105234774 | No Hit |
| CCCCGGGGAGCCCGGCGGGC | 35540 | 0.29176900268773925 | No Hit |
| CCCCACAACCGCGCTTGACTAGCT | 35103 | 0.2881814097171556 | No Hit |
| AGGGCGCCCTGGAAT | 33471 | 0.27478335084303096 | No Hit |
| GGGGGGAGAGAAGGGT | 30232 | 0.24819247296724067 | No Hit |
| TGGCCGCCGGTGAAATACCACTAC | 30065 | 0.24682147061921444 | No Hit |
| CCCCGCGGGGGCGCGCCGGC | 29110 | 0.23898130749127997 | No Hit |
| GGGGGCGGCGCGCGC | 28525 | 0.23417869447573897 | No Hit |
| CGCCGGTGAAATACCACTAC | 28277 | 0.2321427149409455 | No Hit |
| AACCCGGCGGCGGGT | 28273 | 0.23210987656135207 | No Hit |
| GGGCCGCCGGTGAAATACCACT | 27261 | 0.22380176652421102 | No Hit |
| GGCGGGGCGCGGGAC | 26172 | 0.21486151767989622 | No Hit |
| GGCGGGGCGCGGGACATGTGGCGTACGGAAGACCCGC | 24843 | 0.20395096605997487 | No Hit |
| GGAGATGGGCGCCGC | 24729 | 0.20301507224156173 | No Hit |
| CGCGAGGGGGGCCCGGGC | 24485 | 0.20101193108636173 | No Hit |
| GGCGGGGCGCGGGACATGTGGCGT | 24248 | 0.19906625709545026 | No Hit |
| CCCCACAACCGCGCTTGACT | 24069 | 0.1975967396086437 | No Hit |
| GGGCCGCCGGTGAAATACCACTACAG | 23284 | 0.19115220761343055 | No Hit |
| TGGGCCGCCGGTGAAATACCACTAC | 22588 | 0.1854383295641715 | No Hit |
| GGAGAAGCCGGCGGGAGC | 20681 | 0.16978263209299763 | No Hit |
| GGCTGGTCCGATGGTAGTGGGTTATC | 20556 | 0.16875643273070257 | No Hit |
| GGGGGCGGGGAGCGGT | 19962 | 0.16387993336107629 | No Hit |
| GAAGAGGGGGACGGC | 18933 | 0.15543226021066314 | No Hit |
| GGGCCGCCGGTGAAATACCACTACAT | 18926 | 0.1553747930463746 | No Hit |
| CCCGGGGCCGAGGGAGC | 18558 | 0.15235366212377788 | No Hit |
| CCGAGAGAGGGGCCCGT | 18027 | 0.14799436723274834 | No Hit |
| CGGTGAAATACCACTAC | 17283 | 0.14188642862836798 | No Hit |
| GGGAAGGCCCGGCGGGGAAGGT | 16869 | 0.13848765634044666 | No Hit |
| ACCCCCGCGGGGAAT | 15856 | 0.13017133670840725 | No Hit |
| CGGTCGGGCTGGGGCGC | 15451 | 0.12684645077457118 | No Hit |
| GAGGGGGGCCCGGGC | 15414 | 0.12654269576333183 | No Hit |
| GTCTACGGCCATACCACCCTGAACGCGCCCGATCTCGTCTGATCTCGG | 15382 | 0.12627998872658427 | No Hit |
| CCCCACAACCGCGCTTGACTAGCTT | 14457 | 0.11868611344560065 | No Hit |
| GGGTCGGTCGGGCTGGGGCGC | 14330 | 0.11764349489350884 | No Hit |
| GTCCCCCGAAGAGGGGGACGGC | 13496 | 0.110796692748276 | No Hit |
| AGCGCCGAATCCCCGCCCCGC | 13371 | 0.10977049338598092 | No Hit |
| GGGTCGGGGCGGCGGCGGCGGC | 13281 | 0.10903162984512846 | No Hit |
| CCGCGAGGGGGGCCCGGGC | 12951 | 0.10632246352866943 | No Hit |
| GGGGCCGCCGGTGAAATACCACTAC | 12840 | 0.10541119849495138 | No Hit |
| GAAGCCAGAGGAAACTCTGGT | 12630 | 0.10368718356629564 | No Hit |
| GGGCCGCCGGTGAAATACCACG | 12495 | 0.10257888825501693 | No Hit |

## Adapter Content

Produced by FastQC (version 0.11.8)

FastQC Report

Sat 1 Feb 2020  
130b\_SS.fastq

## Summary

- Basic Statistics
- Per base sequence quality
- Per tile sequence quality
- Per sequence quality scores
- Per base sequence content
- Per sequence GC content
- Per base N content
- Sequence Length Distribution
- Sequence Duplication Levels
- Overrepresented sequences
- Adapter Content

## Basic Statistics

| Measure | Value |
| --- | --- |
| Filename | 130b\_SS.fastq |
| File type | Conventional base calls |
| Encoding | Sanger / Illumina 1.9 |
| Total Sequences | 14460935 |
| Sequences flagged as poor quality | 0 |
| Sequence length | 15-48 |
| %GC | 57 |

## Per base sequence quality

## Per tile sequence quality

## Per sequence quality scores

## Per base sequence content

## Per sequence GC content

## Per base N content

## Sequence Length Distribution

## Sequence Duplication Levels

## Overrepresented sequences

| Sequence | Count | Percentage | Possible Source |
| --- | --- | --- | --- |
| GGGCCGCCGGTGAAATACCACTAC | 2646622 | 18.30187328827631 | No Hit |
| GGCCGCCGGTGAAATACCACTAC | 157534 | 1.0893763093465256 | No Hit |
| GGCTGGTCCGATGGTAGTGGGTTATCAGAAC | 136322 | 0.9426914649709718 | No Hit |
| GGGCCGCCGGTGAAATACCACTACG | 131657 | 0.910432140107123 | No Hit |
| GGGCCGCCGGTGAAATACCACTAT | 96788 | 0.6693066527164391 | No Hit |
| CCCCACAACCGCGCTTGACTAGC | 88656 | 0.613072391238879 | No Hit |
| CCCCACAACCGCGCTTGACTAGCTTGCTGTTT | 73765 | 0.5100984134151768 | No Hit |
| CCCCCGCGGGGGCGCGCCGGC | 73181 | 0.5060599470227893 | No Hit |
| TGGCCGCCGGTGAAATACCACTAC | 64372 | 0.4451441072102184 | No Hit |
| AGGGCGCCCTGGAAT | 61744 | 0.4269710084444748 | No Hit |
| GGGCCGCCGGTGAAATACCACTACAG | 60755 | 0.42013189326969524 | No Hit |
| GGGTCGGGGCGGCGGC | 52570 | 0.36353112713666164 | No Hit |
| TGGGCCGCCGGTGAAATACCACTAC | 52530 | 0.3632545198495118 | No Hit |
| GAGAGAGGGGCCCGT | 51494 | 0.35609039111233126 | No Hit |
| GGGCCGCCGGTGAAATACCACTACAT | 50976 | 0.352508326743741 | No Hit |
| GGGGGGAGAGAAGGGT | 49973 | 0.34557239901845904 | No Hit |
| AGGGAAGAGCCCAGCGCCGAATCCCCGCCCCGC | 49449 | 0.3419488435567963 | No Hit |
| CCCCACAACCGCGCTTGACTAGCT | 42016 | 0.2905482944221795 | No Hit |
| GGCGGGGCGCGGGACATGTGGCGT | 40946 | 0.28314904949092157 | No Hit |
| CCGAGAGAGGGGCCCGT | 37160 | 0.25696816976219033 | No Hit |
| GGCTGGTCCGATGGTAGTGGGTTATC | 35452 | 0.2451570386008927 | No Hit |
| GGGCCGCCGGTGAAATACCACTACAAG | 33149 | 0.22923137404324131 | No Hit |
| GGAGATGGGCGCCGC | 32230 | 0.222876321620974 | No Hit |
| CGCCGGTGAAATACCACTAC | 31501 | 0.2178351538126684 | No Hit |
| CCCCGCGGGGGCGCGCCGGC | 29932 | 0.20698523297421637 | No Hit |
| GGGGCCGCCGGTGAAATACCACTAC | 29824 | 0.2062383932989119 | No Hit |
| GAGGCGTCCAGTGCGGTAACGCGAC | 29654 | 0.2050628123285251 | No Hit |
| CCCGGGGCCGAGGGAGC | 24582 | 0.1699890083179269 | No Hit |
| CCCCACTGCTAAATTTGACTGGCT | 24228 | 0.1675410338266509 | No Hit |
| GGGCCGCCTGTGAAATACCACTAC | 22851 | 0.15801882796651806 | No Hit |
| GGGAAGGCCCGGCGGGGAAGGT | 22753 | 0.157341140113001 | No Hit |
| AGCGCCGAATCCCCGCCCCGC | 22719 | 0.15710602391892364 | No Hit |
| CGCGAGGGGGGCCCGGGC | 22624 | 0.1564490816119428 | No Hit |
| GGGCCGCCGGTGAAATACCACT | 22226 | 0.153696839104802 | No Hit |
| GGGTCGGTCGGGCTGGGGCGC | 20681 | 0.1430128826386399 | No Hit |
| GGCTGGTCCGAAGGTAGTGAGTTATCTCAAT | 20664 | 0.1428953245416012 | No Hit |
| CCCCGGGGAGCCCGGCGGGC | 20536 | 0.14201018122272177 | No Hit |
| GGCGGGGCGCGGGACATGTGGCGTACGGAAGACCCGC | 20192 | 0.13963135855323325 | No Hit |
| AACCCGGCGGCGGGT | 19900 | 0.1376121253570395 | No Hit |
| GGGGGCGGCGCGCGC | 19503 | 0.13486679803207746 | No Hit |
| GGGCCGCCGGTGAAATACCACG | 18624 | 0.12878835289695997 | No Hit |
| CGGTGAAATACCACTAC | 18417 | 0.12735691018595963 | No Hit |
| GGCGGGGCGCGGGAC | 17698 | 0.12238489419944146 | No Hit |
| GAAGAGGGGGACGGC | 17639 | 0.12197689845089546 | No Hit |
| GGCTGGTCCGATGGTAGTGGGTTATCAG | 17147 | 0.11857462881895257 | No Hit |
| ACCCCCGCGGGGAAT | 16637 | 0.11504788590779226 | No Hit |
| GGGCCGCCGGTGAAATACCACTACAAAG | 16277 | 0.11255842032344382 | No Hit |
| GGGGGCGGGGAGCGGT | 15808 | 0.10931519988161209 | No Hit |
| GTCCCCCGAAGAGGGGGACGGC | 15578 | 0.10772470798050056 | No Hit |
| GCGGGCCGCCGGTGAAATACCACTAC | 14635 | 0.10120369118594337 | No Hit |

## Adapter Content

Produced by FastQC (version 0.11.8)

FastQC Report

Sat 1 Feb 2020  
134pc\_SS.fastq

## Summary

- Basic Statistics
- Per base sequence quality
- Per tile sequence quality
- Per sequence quality scores
- Per base sequence content
- Per sequence GC content
- Per base N content
- Sequence Length Distribution
- Sequence Duplication Levels
- Overrepresented sequences
- Adapter Content

## Basic Statistics

| Measure | Value |
| --- | --- |
| Filename | 134pc\_SS.fastq |
| File type | Conventional base calls |
| Encoding | Sanger / Illumina 1.9 |
| Total Sequences | 15969710 |
| Sequences flagged as poor quality | 0 |
| Sequence length | 15-48 |
| %GC | 57 |

## Per base sequence quality

## Per tile sequence quality

## Per sequence quality scores

## Per base sequence content

## Per sequence GC content

## Per base N content

## Sequence Length Distribution

## Sequence Duplication Levels

## Overrepresented sequences

| Sequence | Count | Percentage | Possible Source |
| --- | --- | --- | --- |
| GGGCCGCCGGTGAAATACCACTAC | 3004752 | 18.815319752205895 | No Hit |
| GGCCGCCGGTGAAATACCACTAC | 178072 | 1.1150609497605153 | No Hit |
| GGCTGGTCCGATGGTAGTGGGTTATCAGAAC | 176359 | 1.1043343930478389 | No Hit |
| GGGCCGCCGGTGAAATACCACTACG | 106439 | 0.6665055282782217 | No Hit |
| GGGCCGCCGGTGAAAT | 88303 | 0.5529405355513657 | No Hit |
| GGGCCGCCGGTGAAATACCACT | 86924 | 0.5443054382327543 | No Hit |
| CCCCCGCGGGGGCGCGCCGGC | 77375 | 0.48451098986769325 | No Hit |
| CCCCACAACCGCGCTTGACTAGCTTGCTGTTT | 67423 | 0.42219301414991256 | No Hit |
| GGGCCGCCGGTGAAATACCACTAT | 66009 | 0.41333875192473746 | No Hit |
| GGCGGGGCGCGGGACATGTGGCGT | 62643 | 0.39226134976777915 | No Hit |
| GGGCCGCCGGTGAAATACCACTACAG | 61202 | 0.38323801747182634 | No Hit |
| CCCCACAACCGCGCTTGACTAGC | 57446 | 0.3597184920703006 | No Hit |
| GGGTCGGGGCGGCGGC | 54637 | 0.3421289428549423 | No Hit |
| TGGCCGCCGGTGAAATACCACTAC | 53303 | 0.3337756289876272 | No Hit |
| GGGCCGCCGGTGAAATACCACTACAT | 51374 | 0.3216965117087286 | No Hit |
| CGCCGGTGAAATACCACTAC | 42056 | 0.2633485517269882 | No Hit |
| TGGGCCGCCGGTGAAATACCACTAC | 42035 | 0.2632170527830499 | No Hit |
| AGGGCGCCCTGGAAT | 40424 | 0.25312920522664467 | No Hit |
| GAGAGAGGGGCCCGT | 39584 | 0.24786924746911498 | No Hit |
| GGGGGCGGCGCGCGC | 39358 | 0.24645406835816053 | No Hit |
| CCGAGAGAGGGGCCCGT | 36509 | 0.22861404496387225 | No Hit |
| AGGGAAGAGCCCAGCGCCGAATCCCCGCCCCGC | 36475 | 0.22840114191178176 | No Hit |
| CCCCACAACCGCGCTTGACTAGCT | 35979 | 0.22529526209304993 | No Hit |
| GGGGCCGCCGGTGAAATACCACTAC | 35470 | 0.22210797816616581 | No Hit |
| GGGCCGCCGGTGAAATACCACTACAAG | 34244 | 0.21443094458196174 | No Hit |
| GAGGCGTCCAGTGCGGTAACGCGAC | 33636 | 0.21062373706222595 | No Hit |
| CCCCACAACCGCGCTTGACT | 31353 | 0.1963279232997969 | No Hit |
| AGCGCCGAATCCCCGCCCCGC | 30287 | 0.18965278643131278 | No Hit |
| CCCCGCGGGGGCGCGCCGGC | 26851 | 0.1681370544612269 | No Hit |
| CGCGAGGGGGGCCCGGGC | 24491 | 0.15335907790435768 | No Hit |
| GGCTGGTCCGATGGTAGTGGGTTATC | 24207 | 0.15158071123395478 | No Hit |
| GGGGGGAGAGAAGGGT | 23095 | 0.14461752905970113 | No Hit |
| CGGTGAAATACCACTAC | 21710 | 0.13594486061425035 | No Hit |
| GGCGGGGCGCGGGAC | 20212 | 0.12656460261332234 | No Hit |
| GGGTCTTCCCGGAGTCGGGTTGCT | 19791 | 0.12392836188008423 | No Hit |
| GGGAAGGCCCGGCGGGGAAGGT | 18483 | 0.11573785622907366 | No Hit |
| GGGCCGCCGGTGAAATACCACTACAAAG | 16985 | 0.10635759822814567 | No Hit |
| GAAGAGGGGGACGGC | 16225 | 0.10159858882847592 | No Hit |

## Adapter Content

Produced by FastQC (version 0.11.8)

FastQC Report

Sat 1 Feb 2020  
14pc\_SS.fastq

## Summary

- Basic Statistics
- Per base sequence quality
- Per tile sequence quality
- Per sequence quality scores
- Per base sequence content
- Per sequence GC content
- Per base N content
- Sequence Length Distribution
- Sequence Duplication Levels
- Overrepresented sequences
- Adapter Content

## Basic Statistics

| Measure | Value |
| --- | --- |
| Filename | 14pc\_SS.fastq |
| File type | Conventional base calls |
| Encoding | Sanger / Illumina 1.9 |
| Total Sequences | 14015181 |
| Sequences flagged as poor quality | 0 |
| Sequence length | 15-48 |
| %GC | 56 |

## Per base sequence quality

## Per tile sequence quality

## Per sequence quality scores

## Per base sequence content

## Per sequence GC content

## Per base N content

## Sequence Length Distribution

## Sequence Duplication Levels

## Overrepresented sequences

| Sequence | Count | Percentage | Possible Source |
| --- | --- | --- | --- |
| GGGCCGCCGGTGAAATACCACTAC | 1621429 | 11.569090688161644 | No Hit |
| GGGCCGCCGGTGAAAT | 175924 | 1.2552388727623283 | No Hit |
| GGGCCGCCGGTGAAATACCACT | 99515 | 0.7100514791781855 | No Hit |
| GGCCGCCGGTGAAATACCACTAC | 98456 | 0.7024953869664616 | No Hit |
| GGCTGGTCCGATGGTAGTGGGTTATCAGAAC | 86198 | 0.6150330844817488 | No Hit |
| GGGCCGCCGGTGAAATACCACTACG | 84852 | 0.6054292127943264 | No Hit |
| GGGCCGCCGGTGAAATACCACTAT | 61937 | 0.44192793514404133 | No Hit |
| CCCCACAACCGCGCTTGACTAGC | 61010 | 0.4353136787887363 | No Hit |
| GGGTCGGGGCGGCGGC | 58348 | 0.41631998901762307 | No Hit |
| CCCCACAACCGCGCTTGACTAGCTTGCTGTTT | 57724 | 0.4118676740600068 | No Hit |
| CCCCCGCGGGGGCGCGCCGGC | 46532 | 0.3320114096278885 | No Hit |
| CGCCGGTGAAATACCACTAC | 45710 | 0.3261463408856439 | No Hit |
| GGGCCGCCGGTGAAATACCACTACAG | 36420 | 0.2598610749301062 | No Hit |
| GGGGGCGGCGCGCGC | 31292 | 0.22327217893225926 | No Hit |
| TGGCCGCCGGTGAAATACCACTAC | 30716 | 0.21916234974061344 | No Hit |
| GGGCCGCCGGTGAAATACCACTACAT | 30084 | 0.214652953822002 | No Hit |
| AGGGAAGAGCCCAGCGCCGAATCCCCGCCCCGC | 27195 | 0.1940395917826534 | No Hit |
| GGGCCGCCGGTGAAAC | 26949 | 0.19228435223205467 | No Hit |
| CGGTGAAATACCACTAC | 26802 | 0.19123548957377004 | No Hit |
| GGCTGGTCCGATGGTAGTGGGTTATC | 25733 | 0.18360804616080234 | No Hit |
| GGGCCGCCGGTGAAATACC | 23593 | 0.1683388890946182 | No Hit |
| GGGAAGGCCCGGCGGGGAAGGT | 23578 | 0.16823186229275242 | No Hit |
| CCCCACAACCGCGCTTGACTAGCT | 23131 | 0.16504246359715224 | No Hit |
| TGGGCCGCCGGTGAAATACCACTAC | 22478 | 0.16038323015592876 | No Hit |
| GGCTGGTCCGATGGTAGTGGGTT | 21946 | 0.1565873462497559 | No Hit |
| CCCCACAACCGCGCTTGACT | 20784 | 0.14829633666522038 | No Hit |
| GGGCCGCCGGTGAAATACCACTACAAG | 19510 | 0.1392061936267537 | No Hit |
| GGCGGGGCGCGGGACATGTGGCGT | 19270 | 0.13749376479690129 | No Hit |
| CGCCGGTGAAATACCACT | 18758 | 0.1338405832932161 | No Hit |
| GGGGGGAGAGAAGGGT | 18608 | 0.13277031527455835 | No Hit |
| GGGGCCGCCGGTGAAATACCACTAC | 18580 | 0.13257053191107557 | No Hit |
| GGCGGGGCGCGGGAC | 17516 | 0.1249787640987298 | No Hit |
| CCCCGCGGGGGCGCGCCGGC | 16799 | 0.11986288296954567 | No Hit |
| CGCGAGGGGGGCCCGGGC | 16533 | 0.11796494101645923 | No Hit |
| AGGGCGCCCTGGAAT | 16157 | 0.11528213584969041 | No Hit |
| CGGTGAAATACCACT | 15962 | 0.11389078742543532 | No Hit |
| GGCGGGGCGCGGGACATGTGGCGTACGGAAGACCCGC | 15840 | 0.11302030277026034 | No Hit |
| AGCGCCGAATCCCCGCCCCGC | 15339 | 0.1094456075879434 | No Hit |
| GTCTACGGCCATACCACCCTGAACGCGCCCGATCTCGTCTGATCTCGG | 14688 | 0.10480064438696866 | No Hit |
| GGGCCGCCGGTGAAATACCACC | 14563 | 0.10390875437142054 | No Hit |

## Adapter Content

Produced by FastQC (version 0.11.8)

FastQC Report

Sat 1 Feb 2020  
47b\_SS.fastq

## Summary

- Basic Statistics
- Per base sequence quality
- Per tile sequence quality
- Per sequence quality scores
- Per base sequence content
- Per sequence GC content
- Per base N content
- Sequence Length Distribution
- Sequence Duplication Levels
- Overrepresented sequences
- Adapter Content

## Basic Statistics

| Measure | Value |
| --- | --- |
| Filename | 47b\_SS.fastq |
| File type | Conventional base calls |
| Encoding | Sanger / Illumina 1.9 |
| Total Sequences | 15961898 |
| Sequences flagged as poor quality | 0 |
| Sequence length | 15-48 |
| %GC | 56 |

## Per base sequence quality

## Per tile sequence quality

## Per sequence quality scores

## Per base sequence content

## Per sequence GC content

## Per base N content

## Sequence Length Distribution

## Sequence Duplication Levels

## Overrepresented sequences

| Sequence | Count | Percentage | Possible Source |
| --- | --- | --- | --- |
| GGGCCGCCGGTGAAATACCACTAC | 2533366 | 15.871333095851131 | No Hit |
| GGCCGCCGGTGAAATACCACTAC | 150232 | 0.9411913295022936 | No Hit |
| GGGCCGCCGGTGAAATACCACTACG | 143740 | 0.9005194745637392 | No Hit |
| AGGGAAGAGCCCAGCGCCGAATCCCCGCCCCGC | 140854 | 0.8824389179783007 | No Hit |
| GGGCCGCCGGTGAAATACCACTAT | 116494 | 0.7298254881718954 | No Hit |
| CCCCACAACCGCGCTTGACTAGCTTGCTGTTT | 95831 | 0.6003734643586871 | No Hit |
| CCCCACAACCGCGCTTGACTAGC | 93997 | 0.5888836026893544 | No Hit |
| GGCTGGTCCGATGGTAGTGGGTTATCAGAAC | 92048 | 0.5766732753210175 | No Hit |
| GAGAGAGGGGCCCGT | 79868 | 0.5003665604178149 | No Hit |
| AGGGCGCCCTGGAAT | 72935 | 0.4569318761465585 | No Hit |
| TGGCCGCCGGTGAAATACCACTAC | 63398 | 0.39718334248220355 | No Hit |
| GGCGGGGCGCGGGACATGTGGCGTACGGAAGACCCGC | 61338 | 0.3842776090913499 | No Hit |
| GGCGGGGCGCGGGACATGTGGCGT | 59280 | 0.3713844055387398 | No Hit |
| GGGTCGGGGCGGCGGC | 56305 | 0.3527462711514633 | No Hit |
| CCGAGAGAGGGGCCCGT | 52650 | 0.32984799176138074 | No Hit |
| GGGCCGCCGGTGAAATACCACTACAG | 52369 | 0.3280875494881624 | No Hit |
| CCCCCGCGGGGGCGCGCCGGC | 49261 | 0.30861618085769 | No Hit |
| TGGGCCGCCGGTGAAATACCACTAC | 47477 | 0.29743956514444586 | No Hit |
| CCCCACAACCGCGCTTGACTAGCT | 45358 | 0.28416420152540756 | No Hit |
| GGGCCGCCGGTGAAATACCACTACAT | 42018 | 0.2632393716586837 | No Hit |
| GGGGGCGGCGCGCGC | 31481 | 0.19722591887255514 | No Hit |
| GGAGATGGGCGCCGC | 31236 | 0.19569101368772057 | No Hit |
| CGCCGGTGAAATACCACTAC | 28416 | 0.17802394176431902 | No Hit |
| GGGGCCGCCGGTGAAATACCACTAC | 28251 | 0.17699023010922635 | No Hit |
| GGGCCGCCGGTGAAATACCACTACAAG | 27883 | 0.17468473987241367 | No Hit |
| GGGCCGCCGGTGAAATACCACG | 27612 | 0.17298694679041302 | No Hit |
| CCCCGCGGGGGCGCGCCGGC | 27279 | 0.1709007287228624 | No Hit |
| CGCGAGGGGGGCCCGGGC | 25775 | 0.1614782903637149 | No Hit |
| GAAGAGGGGGACGGC | 25272 | 0.15832703604546278 | No Hit |
| CGAGAGAGGGGCCCGT | 24175 | 0.1514544197688771 | No Hit |
| ACCCCCGCGGGGAAT | 23242 | 0.14560925022826232 | No Hit |
| CCCCACAACCGCGCTTGACT | 22387 | 0.1402527443791459 | No Hit |
| GAGAGAGGGGCCCGC | 21555 | 0.13504033166983023 | No Hit |
| AACCCGGCGGCGGGT | 20636 | 0.1292828709969203 | No Hit |
| GGCTGGTCCGATGGTAGTGGGTTATC | 20059 | 0.1256680126636569 | No Hit |
| GGGGGGAGAGAAGGGT | 19862 | 0.12443382359666752 | No Hit |
| CCCCACAACCGCGCTTGACTAGCTT | 19758 | 0.12378227200800306 | No Hit |
| GAGGCGTCCAGTGCGGTAACGCGAC | 19035 | 0.11925273548296074 | No Hit |
| GGCGGGGCGCGGGAC | 18716 | 0.11725422628311497 | No Hit |
| GTCCCCCGAAGAGGGGGACGGC | 18467 | 0.11569426142179333 | No Hit |
| GGGCCGCCGGTGAAATACCACT | 18004 | 0.11279360386841214 | No Hit |
| CGGTGAAATACCACTAC | 17864 | 0.11191651519136385 | No Hit |

## Adapter Content

Produced by FastQC (version 0.11.8)

FastQC Report

Sat 1 Feb 2020  
56pc\_SS.fastq

## Summary

- Basic Statistics
- Per base sequence quality
- Per tile sequence quality
- Per sequence quality scores
- Per base sequence content
- Per sequence GC content
- Per base N content
- Sequence Length Distribution
- Sequence Duplication Levels
- Overrepresented sequences
- Adapter Content

## Basic Statistics

| Measure | Value |
| --- | --- |
| Filename | 56pc\_SS.fastq |
| File type | Conventional base calls |
| Encoding | Sanger / Illumina 1.9 |
| Total Sequences | 18059993 |
| Sequences flagged as poor quality | 0 |
| Sequence length | 15-48 |
| %GC | 57 |

## Per base sequence quality

## Per tile sequence quality

## Per sequence quality scores

## Per base sequence content

## Per sequence GC content

## Per base N content

## Sequence Length Distribution

## Sequence Duplication Levels

## Overrepresented sequences

| Sequence | Count | Percentage | Possible Source |
| --- | --- | --- | --- |
| GGGCCGCCGGTGAAATACCACTAC | 3670326 | 20.322964687749327 | No Hit |
| GGCCGCCGGTGAAATACCACTAC | 213172 | 1.1803548318097354 | No Hit |
| GGCTGGTCCGATGGTAGTGGGTTATCAGAAC | 204517 | 1.1324312251948272 | No Hit |
| GGGCCGCCGGTGAAATACCACTACG | 143615 | 0.7952107179665021 | No Hit |
| CCCCCGCGGGGGCGCGCCGGC | 112137 | 0.620913861926746 | No Hit |
| CCCCACAACCGCGCTTGACTAGCTTGCTGTTT | 87268 | 0.48321170445636386 | No Hit |
| GGGCCGCCGGTGAAATACCACTAT | 87036 | 0.48192709709245185 | No Hit |
| CCCCACAACCGCGCTTGACTAGC | 85913 | 0.4757089329990328 | No Hit |
| GGGCCGCCGGTGAAATACCACTACAG | 78715 | 0.4358528821135202 | No Hit |
| GGGCCGCCGGTGAAATACCACT | 74824 | 0.41430802326446087 | No Hit |
| GGGCCGCCGGTGAAATACCACTACAT | 66892 | 0.37038774046036455 | No Hit |
| TGGCCGCCGGTGAAATACCACTAC | 66783 | 0.3697841964833541 | No Hit |
| GAGAGAGGGGCCCGT | 54770 | 0.30326700569596016 | No Hit |
| GGGCCGCCGGTGAAAT | 53119 | 0.29412525242949983 | No Hit |
| GGCGGGGCGCGGGACATGTGGCGT | 52738 | 0.29201561706031665 | No Hit |
| TGGGCCGCCGGTGAAATACCACTAC | 52061 | 0.2882669998820044 | No Hit |
| GGGTCGGGGCGGCGGC | 49523 | 0.27421383828886314 | No Hit |
| GGGCCGCCGGTGAAATACCACTACAAG | 44649 | 0.24722600944529716 | No Hit |
| CGCCGGTGAAATACCACTAC | 44482 | 0.2463013136273087 | No Hit |
| GGGGCCGCCGGTGAAATACCACTAC | 42792 | 0.23694361343329423 | No Hit |
| GGCTGGTCCGATGGTAGTGGGTTATC | 38491 | 0.21312854329456274 | No Hit |
| AGGGAAGAGCCCAGCGCCGAATCCCCGCCCCGC | 37346 | 0.20678856298560028 | No Hit |
| CCCCGCGGGGGCGCGCCGGC | 36698 | 0.203200521727777 | No Hit |
| GGGGGCGGCGCGCGC | 35153 | 0.19464570113620752 | No Hit |
| CCCCACAACCGCGCTTGACTAGCT | 33742 | 0.18683285203931144 | No Hit |
| CGCGAGGGGGGCCCGGGC | 28575 | 0.1582226526887358 | No Hit |
| CCCCACAACCGCGCTTGACT | 26713 | 0.14791257117320034 | No Hit |
| GGCGGGGCGCGGGACATGTGGCGTACGGAAGACCCGC | 24928 | 0.13802884641206672 | No Hit |
| CGGTGAAATACCACTAC | 24813 | 0.1373920798308172 | No Hit |
| AGCGCCGAATCCCCGCCCCGC | 24623 | 0.1363400306965789 | No Hit |
| GAGGCGTCCAGTGCGGTAACGCGAC | 24411 | 0.1351661653467972 | No Hit |
| GGGCCGCCGGTGAAATACCACTACAAAG | 21944 | 0.12150613790381869 | No Hit |
| GGACACGGACAGGATTGACAGATT | 21406 | 0.11852717772371228 | No Hit |
| CCCCACAACCGCGCTTGACTAGCTT | 20368 | 0.1127796671903472 | No Hit |
| GGCTGGTCCGATGGTAGTGGGTTATCAG | 20098 | 0.11128464999958748 | No Hit |
| GGGAAGGCCCGGCGGGGAAGGT | 19770 | 0.10946848096784977 | No Hit |
| GGCCGTGATCGTATAGTGGTTAGTACTCTGCGTTGTGGCCGCAGCAAC | 19745 | 0.10933005345018683 | No Hit |

## Adapter Content

Produced by FastQC (version 0.11.8)

FastQC Report

Sat 1 Feb 2020  
66c\_SS.fastq

## Summary

- Basic Statistics
- Per base sequence quality
- Per tile sequence quality
- Per sequence quality scores
- Per base sequence content
- Per sequence GC content
- Per base N content
- Sequence Length Distribution
- Sequence Duplication Levels
- Overrepresented sequences
- Adapter Content

## Basic Statistics

| Measure | Value |
| --- | --- |
| Filename | 66c\_SS.fastq |
| File type | Conventional base calls |
| Encoding | Sanger / Illumina 1.9 |
| Total Sequences | 16433431 |
| Sequences flagged as poor quality | 0 |
| Sequence length | 15-48 |
| %GC | 58 |

## Per base sequence quality

## Per tile sequence quality

## Per sequence quality scores

## Per base sequence content

## Per sequence GC content

## Per base N content

## Sequence Length Distribution

## Sequence Duplication Levels

## Overrepresented sequences

| Sequence | Count | Percentage | Possible Source |
| --- | --- | --- | --- |
| GGGCCGCCGGTGAAATACCACTAC | 2803434 | 17.05933471835553 | No Hit |
| CCCCACAACCGCGCTTGACTAGCTTGCTGTTT | 187904 | 1.1434252530710112 | No Hit |
| GGGCCGCCGGTGAAATACCACTACG | 169004 | 1.028415794607955 | No Hit |
| GGCCGCCGGTGAAATACCACTAC | 168821 | 1.0273022109625192 | No Hit |
| GGGCCGCCGGTGAAATACCACTAT | 140882 | 0.8572890226027663 | No Hit |
| AGGGAAGAGCCCAGCGCCGAATCCCCGCCCCGC | 127229 | 0.7742083804654062 | No Hit |
| GGCTGGTCCGATGGTAGTGGGTTATCAGAAC | 114436 | 0.6963609729459417 | No Hit |
| CCCCACAACCGCGCTTGACTAGC | 105585 | 0.6425012524773432 | No Hit |
| AGGGCGCCCTGGAAT | 87476 | 0.5323051528314446 | No Hit |
| GGAGATGGGCGCCGC | 77798 | 0.4734130079105209 | No Hit |
| CCCCCGCGGGGGCGCGCCGGC | 69443 | 0.4225715250820112 | No Hit |
| GGCGGGGCGCGGGACATGTGGCGTACGGAAGACCCGC | 64951 | 0.3952370019383049 | No Hit |
| GGGTCGGGGCGGCGGC | 59770 | 0.3637098059437497 | No Hit |
| GGGCCGCCGGTGAAATACCACTACAG | 54388 | 0.3309594934861746 | No Hit |
| GGCGGGGCGCGGGACATGTGGCGT | 53283 | 0.3242353955178319 | No Hit |
| TGGGCCGCCGGTGAAATACCACTAC | 53266 | 0.3241319478567805 | No Hit |
| CCGAGAGAGGGGCCCGT | 49573 | 0.3016594647824913 | No Hit |
| GGGCCGCCGGTGAAATACCACTACAT | 44842 | 0.2728705892275326 | No Hit |
| GGGCCGCCGGTGAAAT | 42300 | 0.2574021213220781 | No Hit |
| CGCCGGTGAAATACCACTAC | 41448 | 0.25221756795644196 | No Hit |
| CCCCGGGGAGCCCGGCGGGC | 40972 | 0.24932103344700202 | No Hit |
| GGGGGCGGCGCGCGC | 38640 | 0.23513044841335934 | No Hit |
| GGGCCGCCGGTGAAATACCACG | 36756 | 0.22366601350624835 | No Hit |
| GGCTGGTCCGATGGTAGTGGGTTATC | 36317 | 0.22099462978850853 | No Hit |
| GGAGAAGCCGGCGGGAGC | 35541 | 0.2162725483193376 | No Hit |
| GGGGCCGCCGGTGAAATACCACTAC | 30505 | 0.18562770002198567 | No Hit |
| CCCCACAACCGCGCTTGACTAGCTTGCTGTT | 30203 | 0.18378998274918976 | No Hit |
| CCCCCACAACCGCGCTTGACTAGCTTGCTGTTT | 29875 | 0.18179405140655047 | No Hit |
| CCCGGGGCCGAGGGAGC | 29223 | 0.17782652934740165 | No Hit |
| CGCGAGGGGGGCCCGGGC | 28770 | 0.17506995343820775 | No Hit |
| GGGCCGCCGGTGAAATACCACTACAAG | 28530 | 0.17360951587042292 | No Hit |
| AACCCGGCGGCGGGT | 28447 | 0.17310444787823068 | No Hit |
| CCCCGCGGGGGCGCGCCGGC | 27282 | 0.16601524051794173 | No Hit |
| GAGGCGTCCAGTGCGGTAACGCGAC | 27212 | 0.16558927956067118 | No Hit |
| GTCTACGGCCATACCACCCTGAACGCGCCCGATCTCGTCTGATCTCGG | 27168 | 0.16532153267324395 | No Hit |
| GGGCCGCCGGTGAAATACCACT | 26986 | 0.16421403418434044 | No Hit |
| CCCCACAACCGCGCTTGACTAGCT | 25827 | 0.15716133776324615 | No Hit |
| GGGGGCGGGGAGCGGT | 25643 | 0.15604166896127777 | No Hit |
| CGGTGAAATACCACTAC | 25324 | 0.15410050402743042 | No Hit |
| GCGGGCCGCCGGTGAAATACCACTAC | 24856 | 0.15125265077024996 | No Hit |
| GGGAAGGCCCGGCGGGGAAGGT | 23976 | 0.14589771302170557 | No Hit |
| TGGCCGCCGGTGAAATACCACTAC | 23354 | 0.14211274565852985 | No Hit |
| GAAGAGGGGGACGGC | 21519 | 0.13094648342150828 | No Hit |
| GGGTCGGTCGGGCTGGGGCGC | 20302 | 0.12354084792153264 | No Hit |
| CCCACAACCGCGCTTGACTAGCTTGCTGTTT | 19115 | 0.11631776711753011 | No Hit |
| CCCCACTGCTAAATTTGACTGGCT | 19042 | 0.11587355069066223 | No Hit |
| GGCGGGGCGCGGGAC | 18724 | 0.11393847091334731 | No Hit |
| GGCTGGTCCGATGGTAGTGGGTTATCAG | 18427 | 0.11213117942321356 | No Hit |
| CCCCCGCGGGGGCGCGCCGGCGT | 17926 | 0.1090825160004627 | No Hit |
| ACCCCCGCGGGGAAT | 17837 | 0.10854093706907583 | No Hit |
| GGGCCGCCTGTGAAATACCACTAC | 17684 | 0.10760990811961299 | No Hit |
| GTCCCCCGAAGAGGGGGACGGC | 17588 | 0.10702573309249906 | No Hit |
| CGACTCTTAGCGGTGGATCACTCGGCTCGTGCGTCGATGAAGAACGCA | 17351 | 0.10558355099431152 | No Hit |

## Adapter Content

Produced by FastQC (version 0.11.8)

FastQC Report

Sat 1 Feb 2020  
67c\_SS.fastq

## Summary

- Basic Statistics
- Per base sequence quality
- Per tile sequence quality
- Per sequence quality scores
- Per base sequence content
- Per sequence GC content
- Per base N content
- Sequence Length Distribution
- Sequence Duplication Levels
- Overrepresented sequences
- Adapter Content

## Basic Statistics

| Measure | Value |
| --- | --- |
| Filename | 67c\_SS.fastq |
| File type | Conventional base calls |
| Encoding | Sanger / Illumina 1.9 |
| Total Sequences | 12870252 |
| Sequences flagged as poor quality | 0 |
| Sequence length | 15-48 |
| %GC | 59 |

## Per base sequence quality

## Per tile sequence quality

## Per sequence quality scores

## Per base sequence content

## Per sequence GC content

## Per base N content

## Sequence Length Distribution

## Sequence Duplication Levels

## Overrepresented sequences

| Sequence | Count | Percentage | Possible Source |
| --- | --- | --- | --- |
| GGGCCGCCGGTGAAATACCACTAC | 594708 | 4.620795303774938 | No Hit |
| GGGTCGGGGCGGCGGC | 43262 | 0.33613949439373836 | No Hit |
| CCGAGAGAGGGGCCCGT | 40075 | 0.31137696449144897 | No Hit |
| GGCCGCCGGTGAAATACCACTAC | 36494 | 0.28355311146976764 | No Hit |
| CCCGGGGCCGAGGGAGC | 35573 | 0.27639707443179823 | No Hit |
| CCCCACAACCGCGCTTGACTAGCTTGCTGTTT | 33129 | 0.2574075472648088 | No Hit |
| AGGGCGCCCTGGAAT | 31408 | 0.24403562572045986 | No Hit |
| CCCCCGCGGGGGCGCGCCGGC | 30997 | 0.2408422150553074 | No Hit |
| CCCCGGGGAGCCCGGCGGGC | 29717 | 0.23089679984510014 | No Hit |
| GGGCCGCCGGTGAAATACCACTACG | 29523 | 0.22938944785230314 | No Hit |
| GTCTACGGCCATACCACCCTGAACGCGCCCGATCTCGTCTGATCTCGG | 28441 | 0.2209824640574248 | No Hit |
| GGCTGGTCCGATGGTAGTGGGTTATCAGAAC | 27533 | 0.21392743514268409 | No Hit |
| CCCCACAACCGCGCTTGACTAGC | 25528 | 0.1983488745985704 | No Hit |
| CGACTCTTAGCGGTGGATCACTCGGCTCGTGCGTCGATGAAGAACGCA | 24633 | 0.19139485380705834 | No Hit |
| AACCCGGCGGCGGGT | 22694 | 0.17632910373472097 | No Hit |
| CGCGAGGGGGGCCCGGGC | 22644 | 0.17594061095307223 | No Hit |
| GGGGGCGGCGCGCGC | 22359 | 0.17372620209767453 | No Hit |
| GGGCCGCCGGTGAAATACCACTAT | 22170 | 0.17225769938304236 | No Hit |
| GGGGGCGGGGAGCGGT | 22129 | 0.17193913530209043 | No Hit |
| GAAGAGGGGGACGGC | 20075 | 0.155979851831961 | No Hit |
| GAAGCCAGAGGAAACTCTGGT | 19181 | 0.14903360089608192 | No Hit |
| GGGCCGCCGGTGAAAT | 19141 | 0.14872280667076293 | No Hit |
| GGAGATGGGCGCCGC | 17866 | 0.1388162407387206 | No Hit |
| CCCCGCGGGGGCGCGCCGGC | 17440 | 0.1355062822390735 | No Hit |
| GTCCCCCGAAGAGGGGGACGGC | 17053 | 0.1324993481091124 | No Hit |
| GAGGCGTCCAGTGCGGTAACGCGAC | 16822 | 0.1307045114578953 | No Hit |
| GGGGGGAGAGAAGGGT | 16326 | 0.12685066306394 | No Hit |
| GGGTCGGTCGGGCTGGGGCGC | 15486 | 0.12032398433224152 | No Hit |
| GGGTGCGATCATACCAGCACTAATGCACCGGATCCCATCAGAACTCCG | 15264 | 0.1185990763817212 | No Hit |
| ACCCCCGCGGGGAAT | 13633 | 0.10592644184433996 | No Hit |
| GGGCCGCCGGTGAAATACCACTACAG | 13460 | 0.10458225681983539 | No Hit |
| CGCCGGTGAAATACCACTAC | 13157 | 0.10222799056304413 | No Hit |

## Adapter Content

Produced by FastQC (version 0.11.8)

FastQC Report

Sat 1 Feb 2020  
7b\_SS.fastq

## Summary

- Basic Statistics
- Per base sequence quality
- Per tile sequence quality
- Per sequence quality scores
- Per base sequence content
- Per sequence GC content
- Per base N content
- Sequence Length Distribution
- Sequence Duplication Levels
- Overrepresented sequences
- Adapter Content

## Basic Statistics

| Measure | Value |
| --- | --- |
| Filename | 7b\_SS.fastq |
| File type | Conventional base calls |
| Encoding | Sanger / Illumina 1.9 |
| Total Sequences | 10488986 |
| Sequences flagged as poor quality | 0 |
| Sequence length | 15-48 |
| %GC | 58 |

## Per base sequence quality

## Per tile sequence quality

## Per sequence quality scores

## Per base sequence content

## Per sequence GC content

## Per base N content

## Sequence Length Distribution

## Sequence Duplication Levels

## Overrepresented sequences

| Sequence | Count | Percentage | Possible Source |
| --- | --- | --- | --- |
| GGGCCGCCGGTGAAATACCACTAC | 612640 | 5.840793380790097 | No Hit |
| CCCCCGCGGGGGCGCGCCGGC | 90225 | 0.8601880105474448 | No Hit |
| CCCCGGGGAGCCCGGCGGGC | 68863 | 0.6565267605467296 | No Hit |
| GGGTCGGGGCGGCGGC | 63691 | 0.6072178950377091 | No Hit |
| AACCCGGCGGCGGGT | 46190 | 0.44036668558810166 | No Hit |
| CCCCGCGGGGGCGCGCCGGC | 45460 | 0.4334070042614224 | No Hit |
| GGGGGCGGGGAGCGGT | 42153 | 0.40187869447056185 | No Hit |
| GGCTGGTCCGATGGTAGTGGGTTATCAGAAC | 41189 | 0.3926881015953306 | No Hit |
| GAGAGAGGGGCCCGT | 39757 | 0.3790356856229954 | No Hit |
| GAAGCCAGAGGAAACTCTGGT | 38854 | 0.3704266551599935 | No Hit |
| CCCGGGGCCGAGGGAGC | 38601 | 0.36801460122074714 | No Hit |
| GGCCGCCGGTGAAATACCACTAC | 36333 | 0.3463919200578588 | No Hit |
| CCCCACAACCGCGCTTGACTAGCTTGCTGTTT | 32846 | 0.3131475244604197 | No Hit |
| AGGGCGCCCTGGAAT | 31196 | 0.2974167379001173 | No Hit |
| GTCTACGGCCATACCACCCTGAACGCGCCCGATCTCGTCTGATCTCGG | 30450 | 0.2903045156128533 | No Hit |
| GGAGATGGGCGCCGC | 30075 | 0.28672933684914825 | No Hit |
| GGGCCGCCGGTGAAATACCACTACG | 29776 | 0.2838787276482207 | No Hit |
| GGGGGGGAGAAGGGT | 27844 | 0.2654594066576121 | No Hit |
| GGGGGCGGCGCGCGC | 27087 | 0.2582423124599461 | No Hit |
| AGGGAAGAGCCCAGCGCCGAATCCCCGCCCCGC | 26809 | 0.25559191326978603 | No Hit |
| CCCCACAACCGCGCTTGACTAGC | 26463 | 0.2522932149971408 | No Hit |
| CCGAGAGAGGGGCCCGT | 26263 | 0.2503864529898314 | No Hit |
| GGCGGGGCGCGGGAC | 25163 | 0.23989926194962985 | No Hit |
| ACCCCCGCGGGGAAT | 24657 | 0.2350751540711371 | No Hit |
| GGGTCGGTCGGGCTGGGGCGC | 23694 | 0.22589409500594243 | No Hit |
| GGGTCGGGGCGGCGGCGGCGGC | 23286 | 0.2220043005110313 | No Hit |
| GAGGCGTCCAGTGCGGTAACGCGAC | 22342 | 0.21300438383653103 | No Hit |
| GGGCCGCCGGTGAAATACCACTAT | 21665 | 0.20654999444178873 | No Hit |
| CCGCGAGGGGGGCCCGGGC | 21423 | 0.2042428124129444 | No Hit |
| GGGCCGCCGGTGAAAT | 21394 | 0.20396633192188454 | No Hit |
| GGCCGGGGGGCGGGCGC | 21341 | 0.20346103998994755 | No Hit |
| CGCGAGGGGGGCCCGGGC | 20679 | 0.1971496577457535 | No Hit |
| GAAGAGGGGGACGGC | 18553 | 0.17688077760805476 | No Hit |
| GGCCCCGCCGGGGTCGGC | 17777 | 0.16948254101969437 | No Hit |
| CGCCGGTGAAATACCACTAC | 17303 | 0.16496351506237114 | No Hit |
| GTCCCCCGAAGAGGGGGACGGC | 17266 | 0.1646107640910189 | No Hit |
| CCCGCCGGGGTCGGC | 16957 | 0.1616648167897259 | No Hit |
| GAATCCCCGCCCCGC | 16536 | 0.15765108276433967 | No Hit |
| GGGAAGGCCCGGCGGGGAAGGT | 16246 | 0.15488627785374107 | No Hit |
| CGGTCGGGCTGGGGCGC | 15666 | 0.14935666803254385 | No Hit |
| TGGCCGCCGGTGAAATACCACTAC | 15622 | 0.14893718039093579 | No Hit |
| GAAGCCAGAGGAAACTCTGGTGGAGGT | 15208 | 0.14499018303580538 | No Hit |
| GGGGAGCCCGGCGGGC | 14569 | 0.13889807842245191 | No Hit |
| GGAGAAGCCGGCGGGAGC | 14463 | 0.1378874945585779 | No Hit |
| CGACTCTTAGCGGTGGATCACTCGGCTCGTGCGTCGATGAAGAACGCA | 14183 | 0.1352180277483448 | No Hit |
| CCCCACTGCTAAATTTGACTGGCT | 13606 | 0.12971701935725724 | No Hit |
| GGGCCGCCGGTGAAATACCACTACAG | 12923 | 0.12320542710229569 | No Hit |
| GGGCCGCCGGTGAAATACCACT | 12669 | 0.12078383935301276 | No Hit |
| GGCGGGGCGCGGGACATGTGGCGT | 12655 | 0.1206503660125011 | No Hit |
| AGCCCCTCTCCGGCC | 12556 | 0.11970651881888297 | No Hit |
| GGCGGGGCGCGGGACATGTGGCGTACGGAAGACCCGC | 12422 | 0.11842898827398568 | No Hit |
| CCCCACAACCGCGCTTGACTAGCT | 11626 | 0.11084007548489434 | No Hit |
| TGGGCCGCCGGTGAAATACCACTAC | 11435 | 0.10901911776791388 | No Hit |
| GGGGGCGGGGAGCGGC | 11325 | 0.10797039866389373 | No Hit |
| GAGAGAGGGGCCCGC | 11075 | 0.105586946154757 | No Hit |
| GGCTGGTCCGATGGTAGTGGGTTATC | 10945 | 0.1043475508500059 | No Hit |
| CCCCGGTGTCCCCGC | 10890 | 0.10382319129799583 | No Hit |
| GGGCCGCCGGTGAAATACCACTACAT | 10610 | 0.10115372448776269 | No Hit |

## Adapter Content

Produced by FastQC (version 0.11.8)

FastQC Report

Sat 1 Feb 2020  
97c\_SS.fastq

## Summary

- Basic Statistics
- Per base sequence quality
- Per tile sequence quality
- Per sequence quality scores
- Per base sequence content
- Per sequence GC content
- Per base N content
- Sequence Length Distribution
- Sequence Duplication Levels
- Overrepresented sequences
- Adapter Content

## Basic Statistics

| Measure | Value |
| --- | --- |
| Filename | 97c\_SS.fastq |
| File type | Conventional base calls |
| Encoding | Sanger / Illumina 1.9 |
| Total Sequences | 19118771 |
| Sequences flagged as poor quality | 0 |
| Sequence length | 15-48 |
| %GC | 57 |

## Per base sequence quality

## Per tile sequence quality

## Per sequence quality scores

## Per base sequence content

## Per sequence GC content

## Per base N content

## Sequence Length Distribution

## Sequence Duplication Levels

## Overrepresented sequences

| Sequence | Count | Percentage | Possible Source |
| --- | --- | --- | --- |
| GGGCCGCCGGTGAAATACCACTAC | 4424572 | 23.14255450834157 | No Hit |
| GGCCGCCGGTGAAATACCACTAC | 261116 | 1.3657572445425492 | No Hit |
| GGGCCGCCGGTGAAATACCACTACG | 178821 | 0.9353163966449517 | No Hit |
| GGCTGGTCCGATGGTAGTGGGTTATCAGAAC | 119483 | 0.6249512586347731 | No Hit |
| CCCCCGCGGGGGCGCGCCGGC | 104208 | 0.5450559557410882 | No Hit |
| GGGCCGCCGGTGAAATACCACTAT | 101411 | 0.5304263542881495 | No Hit |
| GGGCCGCCGGTGAAATACCACTACAG | 97621 | 0.5106029043394055 | No Hit |
| TGGGCCGCCGGTGAAATACCACTAC | 84146 | 0.440122432555942 | No Hit |
| GGGCCGCCGGTGAAATACCACTACAT | 80351 | 0.4202728302985584 | No Hit |
| GGGCCGCCGGTGAAATACCACT | 71620 | 0.3746056689522564 | No Hit |
| GGGCCGCCTGTGAAATACCACTAC | 68596 | 0.3587887526870843 | No Hit |
| CGCCGGTGAAATACCACTAC | 64870 | 0.3393000522889259 | No Hit |
| GAGGCGTCCAGTGCGGTAACGCGAC | 55554 | 0.2905730708318019 | No Hit |
| GGGCCGCCGGTGAAATACCACTACAAG | 55404 | 0.28978850157261676 | No Hit |
| GGCGGGGCGCGGGACATGTGGCGT | 53741 | 0.2810902437191177 | No Hit |
| GGGGCCGCCGGTGAAATACCACTAC | 50580 | 0.26455675419722324 | No Hit |
| CCCCACAACCGCGCTTGACTAGC | 47938 | 0.2507378743121093 | No Hit |
| GGGCCGCCGGTGAAAT | 47389 | 0.24786635082349176 | No Hit |
| CGGTGAAATACCACTAC | 39367 | 0.2059075868422714 | No Hit |
| AGGGAAGAGCCCAGCGCCGAATCCCCGCCCCGC | 38346 | 0.20056728541808466 | No Hit |
| CCCCACAACCGCGCTTGACTAGCTTGCTGTTT | 36147 | 0.18906550007843076 | No Hit |
| GGGGGCGGCGCGCGC | 35992 | 0.18825477851060612 | No Hit |
| CCGAGAGAGGGGCCCGT | 35545 | 0.1859167621182345 | No Hit |
| TGGCCGCCGGTGAAATACCACTAC | 35217 | 0.184201170671483 | No Hit |
| AGGGCGCCCTGGAAT | 34852 | 0.18229205214079922 | No Hit |
| CCCCGCGGGGGCGCGCCGGC | 31185 | 0.16311194898458692 | No Hit |
| GGGTCGGGGCGGCGGC | 30803 | 0.16111391260452881 | No Hit |
| GGTCCGCCGGTGAAATACCACTAC | 30663 | 0.16038164796262272 | No Hit |
| GGCTGGTCCGATGGTAGTGGGTTATC | 29238 | 0.15292824000036404 | No Hit |
| GGACACGGACAGGATTGACAGAT | 28010 | 0.14650523299850185 | No Hit |
| GGGCCGCCGGTGAAATACCACTACAAAG | 27327 | 0.14293282763834558 | No Hit |
| AGCGCCGAATCCCCGCCCCGC | 23687 | 0.12389394694878662 | No Hit |
| GGGTCTTCCCGGAGTCGGGTTGCT | 21809 | 0.11407113982378889 | No Hit |
| GGTGAAATACCACTAC | 21402 | 0.11194234190053325 | No Hit |
| CGCGAGGGGGGCCCGGGC | 20545 | 0.10745983619972226 | No Hit |
| GGGCCGCCGGTGAAATACC | 19737 | 0.1032336231235784 | No Hit |
| GGCCGTGATCGTATAGTGGTTAGTACTCTGCGTTGTGGCCGCAGCAAC | 19689 | 0.10298256096063915 | No Hit |
| GGGCCGCCGGTGAAATACCACTACAAT | 19425 | 0.10160171906447334 | No Hit |

## Adapter Content

Produced by FastQC (version 0.11.8)

FastQC Report

Sat 1 Feb 2020  
98c\_SS.fastq

## Summary

- Basic Statistics
- Per base sequence quality
- Per tile sequence quality
- Per sequence quality scores
- Per base sequence content
- Per sequence GC content
- Per base N content
- Sequence Length Distribution
- Sequence Duplication Levels
- Overrepresented sequences
- Adapter Content

## Basic Statistics

| Measure | Value |
| --- | --- |
| Filename | 98c\_SS.fastq |
| File type | Conventional base calls |
| Encoding | Sanger / Illumina 1.9 |
| Total Sequences | 13274977 |
| Sequences flagged as poor quality | 0 |
| Sequence length | 15-48 |
| %GC | 58 |

## Per base sequence quality

## Per tile sequence quality

## Per sequence quality scores

## Per base sequence content

## Per sequence GC content

## Per base N content

## Sequence Length Distribution

## Sequence Duplication Levels

## Overrepresented sequences

| Sequence | Count | Percentage | Possible Source |
| --- | --- | --- | --- |
| GGGCCGCCGGTGAAATACCACTAC | 3090783 | 23.28277480254768 | No Hit |
| GGCCGCCGGTGAAATACCACTAC | 182353 | 1.3736596304460642 | No Hit |
| GGGCCGCCGGTGAAATACCACTACG | 133066 | 1.0023821510199227 | No Hit |
| GGCTGGTCCGATGGTAGTGGGTTATCAGAAC | 103909 | 0.7827433523990287 | No Hit |
| GGGCCGCCGGTGAAATACCACTAT | 85509 | 0.6441367092387429 | No Hit |
| GGGCCGCCGGTGAAATACCACTACAG | 69708 | 0.5251082544248475 | No Hit |
| CCCCCGCGGGGGCGCGCCGGC | 60911 | 0.45884071964870443 | No Hit |
| CCCCACAACCGCGCTTGACTAGC | 58136 | 0.43793672862860705 | No Hit |
| GGGCCGCCGGTGAAATACCACTACAT | 57613 | 0.4339969854561707 | No Hit |
| GGCGGGGCGCGGGACATGTGGCGT | 57007 | 0.42943200579556556 | No Hit |
| CCCCACAACCGCGCTTGACTAGCTTGCTGTTT | 55540 | 0.4183811391914276 | No Hit |
| CGCCGGTGAAATACCACTAC | 54150 | 0.4079103112570364 | No Hit |
| TGGGCCGCCGGTGAAATACCACTAC | 52639 | 0.3965279939844717 | No Hit |
| AGGGCGCCCTGGAAT | 46621 | 0.35119458210737386 | No Hit |
| AGGGAAGAGCCCAGCGCCGAATCCCCGCCCCGC | 46509 | 0.350350889496833 | No Hit |
| GGGCCGCCGGTGAAAT | 43772 | 0.3297331513267405 | No Hit |
| GGGCCGCCGGTGAAATACCACT | 42158 | 0.31757493817126764 | No Hit |
| GAGGCGTCCAGTGCGGTAACGCGAC | 40718 | 0.3067274617500279 | No Hit |
| GGGCCGCCGGTGAAATACCACTACAAG | 39039 | 0.2940796055616518 | No Hit |
| GGAGATGGGCGCCGC | 36654 | 0.2761134727389735 | No Hit |
| GGGGCCGCCGGTGAAATACCACTAC | 35406 | 0.26671232650723237 | No Hit |
| GGGCCGCCTGTGAAATACCACTAC | 34946 | 0.2632471604282252 | No Hit |
| GGGGGCGGCGCGCGC | 30804 | 0.23204559977768702 | No Hit |
| AGCGCCGAATCCCCGCCCCGC | 30186 | 0.22739022448023827 | No Hit |
| CCGAGAGAGGGGCCCGT | 29788 | 0.2243921025249234 | No Hit |
| CGGTGAAATACCACTAC | 28751 | 0.21658041290768337 | No Hit |
| GGGTCGGGGCGGCGGC | 27578 | 0.20774423940621514 | No Hit |
| TGGCCGCCGGTGAAATACCACTAC | 25109 | 0.18914533712563117 | No Hit |
| GGCTGGTCCGATGGTAGTGGGTTATC | 24794 | 0.186772451658485 | No Hit |
| GGGTCGGTCGGGCTGGGGCGC | 24372 | 0.18359353842948278 | No Hit |
| CCCCACAACCGCGCTTGACT | 23471 | 0.17680633269647097 | No Hit |
| GGGCCGCCGGTGAAATACCACTACAAAG | 19396 | 0.14610948101830987 | No Hit |
| CCCCGGGGAGCCCGGCGGGC | 19339 | 0.14568010174330245 | No Hit |
| GGAGAAGCCGGCGGGAGC | 19308 | 0.14544657968145633 | No Hit |
| GGCGGGGCGCGGGACATGTGGCGTACGGAAGACCCGC | 19236 | 0.14490420586039432 | No Hit |
| CCCCGCGGGGGCGCGCCGGC | 19193 | 0.14458028816170454 | No Hit |
| GGTCCGCCGGTGAAATACCACTAC | 18774 | 0.14142397384191324 | No Hit |
| CCCCACAACCGCGCTTGACTAGCT | 17893 | 0.13478742750364087 | No Hit |
| GGGGGCGGGGAGCGGT | 17647 | 0.13293431694834573 | No Hit |
| CCCCACTGCTAAATTTGACTGGCT | 17615 | 0.13269326191676264 | No Hit |
| GGGAAGGCCCGGCGGGGAAGGT | 16740 | 0.1261019133969121 | No Hit |
| GAAGAGGGGGACGGC | 16468 | 0.1240529456284557 | No Hit |
| CCCGGGGCCGAGGGAGC | 16261 | 0.1224936208929025 | No Hit |
| CGCGAGGGGGGCCCGGGC | 15190 | 0.11442581030460543 | No Hit |
| GGGCCGCCGGTGAAATACCACG | 14869 | 0.11200772701903738 | No Hit |
| GGGCCGCCGGTGAAATACCACTACAAT | 14385 | 0.10836176966634292 | No Hit |
| GGGCCGCCGGTGAAATACCACTACT | 13527 | 0.1018984816320209 | No Hit |

## Adapter Content

Produced by FastQC (version 0.11.8)
